# Social Media Influencer Viewing and Intentions to Change Appearance: A Large Scale Cross-Sectional Survey on Female Social Media Users in China

**DOI:** 10.3389/fpsyg.2022.846390

**Published:** 2022-04-08

**Authors:** Wenjing Pan, Zhe Mu, Zheng Tang

**Affiliations:** School of Journalism and Communication, Renmin University of China, Beijing, China

**Keywords:** social media influencer, influencer viewing, self-objectification, social comparison tendencies, intentions to change appearance, TikTok

## Abstract

Previous studies have reported that general or photo-specific social media use was associated with women’s body dissatisfaction and body image disturbance. The current study replicated and expanded upon these findings by identifying the positive association between social media influencer viewing and intentions to change appearance. This study surveyed a sample of 7,015 adult female TikTok users in China regarding their social media influencer viewing frequency, self-objectification, social comparison tendencies when watching short videos, intentions to change appearance, and demographics. The results showed that female TikTok users’ self-objectification mediated the association between their influencer viewing frequency and their intentions to change appearance. Furthermore, social comparison tendencies moderated the association between influencer viewing and intentions to change appearance in that the proposed association was stronger for female TikTok users who had lower social comparison tendencies when watching short videos, compared to female TikTok users who had higher social comparison tendencies. The counter-intuitive finding on social comparison tendencies indicated that women who have higher social comparison tendencies may be more aware of the negative influences and adjust their expectations. The observed association between social media influencer viewing and intentions to change appearance was statistically significant but trivial in terms of effect size. Although the result could warn policymakers and practitioners to design media and health literacy campaigns to cultivate body positivity, caution should be exercised when evaluating the practical implications.

## Introduction

Unattainable body ideals portrayed by media exert great pressure on women and contribute to a variety of body image concerns. Thus, exposure to idealized media images has been associated with negative body image, especially among women (Grabe et al., 2008). Early studies mainly focus on the association between traditional media exposure such as TV or magazines and body dissatisfaction. Considering the ubiquity of social media sites in modern life, many studies have been shifted to study the relations between social media use and body image. Popular social media sites and applications are featured with photos and videos of individuals, and users may encounter various forms of idealized images which may be linked to a greater level of body dissatisfaction. For example, Internet use, especially time spent on social media is positively associated with negative mood (Fardouly et al., 2015a), body dissatisfaction (Tiggemann and Miller, 2010), and disordered eating behaviors (Murray et al., 2016). One study finds that the use of appearance-related social networking site features such as photo sharing and retouching rather than the overall use was significantly correlated with weight dissatisfaction and drive for thinness (Meier and Gray, 2014). Instagram use is associated with body dissatisfaction since it exposed individuals to attractive celebrities, models, social media influencers, and attractive peers to make comparisons (Cohen et al., 2019). A recent meta-analysis reveals a small and positive effect size for the relationship between social media use (including both general use and appearance-focused use) and body image disturbance (Saiphoo and Vahedi, 2019).

### Social Media Influencer Viewing and Intentions to Change Appearance

Different types of social media and related activities may have different effects on individuals’ body images. Prior studies on social media use and body image mainly focus on Facebook and Instagram, given that the former remains one of the most widely used social media sites worldwide while the latter features various photo-based activities such as sharing and retouching. In addition to Facebook and Instagram, other emerging sites or applications which are more popular among young people receive rare attention. TikTok, a smartphone-based application with the function of viewing, creating, and sharing short videos, is one of the fastest-growing social media in the world. A Pew Research Center survey reports that 21% of Americans have used TikTok (Auxier and Anderson, 2021), and 62% of TikTok users in the United States are aged between 10 and 29 (Statista, 2021). Given the rapid development of TikTok and other short video sharing applications (e.g., *RED, Kwai* in China) and the high intensity of user engagement, an increased focus on the effects of TikTok usage on body image is warranted. On the one hand, the increasing popularity of short video applications and the high intensity of user engagement are thought to create a higher risk of idealized body image exposure for TikTok users. On the other hand, TikTok and other short video applications also enable users to edit or filter their short videos in order to present an idealized version of themselves. Despite that TikTok use may have parallel negative effects as Facebook and Instagram, as yet there has been little research focusing on the effects of TikTok usage on body image.

In terms of specific targets served as stimuli, studies have examined the different exposure effects of viewing idealized images of models, celebrities, or peers (Lin and Kulik, 2002; Brown and Tiggemann, 2016; Fardouly et al., 2018). Studies report that exposure to both fashion models and celebrity images led to emotional distress and body dissatisfaction (Pan and Peña, 2017, 2020). Similarly, viewing unknown peer images also had a detrimental effect on women’s mood and body image (Brown and Tiggemann, 2016). Women from China and Croatia both reported feeling pressure to conform to standard beauty norms from their family, friends, and media (Stojcic et al., 2020). However, influencers who are highly active on social media have yet to receive enough attention. Social media influencers (hereafter, *influencers*), or micro-celebrities, can be defined as content creators who have a status of expertise in a specific area and have cultivated a sizeable number of captive followers (Lou and Yuan, 2019). Influencers are playing important roles in marketing strategies because of their ability to attract attention and cultivate influence through the practice of self-presentation on social media (Khamis et al., 2017; Lou et al., 2019).

Utilizing images, videos, or other self-presentation strategies, influencers can reinforce their self-branding and commodify their public identities. However, the “authentic self” they presented on social media is more likely to be determined by external recognition rather than their intrinsic motivation (Khamis et al., 2017). A content analysis on 1,000 videos on TikTok reveals that beauty-related content is most common for female influencers on TikTok (Shutsko, 2020). Thus, most content generated by influencers is prone to endorse the social beauty standards and shows a self-objectified tendency. For example, RED is a content-sharing site similar to Instagram where users can post text, images, and short videos, and receive feedback in the form of likes and replies. One experimental study manipulates the social media metrics (such as the number of followers, likes, etc.) of influencer images on RED and finds that social media metrics did not affect female participants’ body satisfaction or mood (Zhang et al., 2021). As a result, this study focuses on a specific media content exposure, that is social media influencer viewing, considering the popularity of influencers on short video applications such as TikTok and their ability to wield influence upon followers.

This study also extends the studies on social media and body image to intentions to change one’s appearance. Most body image-related studies have adopted body (dis)satisfaction, body image concerns, weight concerns, drive for thinness, and body esteem as outcome variables (Want, 2009), while few studies have adopted behavioral intentions, such as intentions to change appearance as outcome variables. Psychological states and perceptions about one’s own body and appearance are worth studying due to their strong associations with individuals’ psychological and physical well-being. However, the behavioral aspects should also be taken into consideration. First, the concept of behavioral intentions was proposed by the theory of planned behaviors and has been studied extensively due to their significant effects in predicting individuals’ various behaviors (Ajzen and Fishbein, 2000). Several studies report a moderate or strong association between intention and actual dieting behavior (Nejad et al., 2004, 2005). One meta-analysis on health-related behavior also shows a similar effect between intention and actual behavior (McEachan et al., 2011). Examining the relationship between social media influencer viewing and intentions to change appearance not only can extend the impact of social media use on body image from psychological aspects to behavioral aspects, but also posits practical implications. Second, relative to body dissatisfaction, which is construed as a negative outcome, intentions to change appearance represent a subjective intent and are not necessarily a positive or negative consequence from exposure to social media influencers. Therefore, we focus on women’s intentions to change appearance as the outcome variable and test whether social media influencer viewing is associated with intentions to change appearance.

### The Mediating Role of Self-Objectification and the Moderating Role of Social Comparison

Several theoretical frameworks have been evoked to explain why exposure to traditional as well as social media may affect individuals’ body image, namely, social comparison theory, thin-ideal internalization, and objectification theory. In the current study, we focus on the objectification theory considering the features of short video applications and characteristics of social media influencers. Objectification theory posits that when women are exposed to sexually objectifying images with a focus on body or appearance, they may in turn view their own bodies as objects to be gazed upon (Fredrickson and Roberts, 1997). Specifically, when been exposed to thin-ideal images or other types of body ideal images focusing on body and appearance, people may also adopt the observers’ point of view and see their own bodies as an “object” (Fredrickson and Roberts, 1997). Self-objectification has been found to be associated with a variety of detrimental body image outcomes among young women, such as body dissatisfaction (Strelan and Hargreaves, 2005), depressed mood, and eating disorders (Tiggemann and Williams, 2012).

Previous research has shown that exposure to thin-ideal fashion magazine advertisements was related to increased self-objectification and body dissatisfaction among young women (Harper and Tiggemann, 2008). Consistent with this, exposure to social media has been associated with self-surveillance and self-objectification (Melioli et al., 2015). For example, research has revealed a link between both Facebook and Instagram usage and young women’s self-objectification (Fardouly et al., 2015b,^2018^). This observed link indicates that experiencing self-objectification through media consumption has already become a part of women’s daily experience.

Furthermore, self-objectification is especially relevant in the context of social media influencer viewing. The content created by female social media influencers is mostly featured with beauty, make-ups, outfits, and fashion topics (Shutsko, 2020). As women may engage in self-objectification indirectly by viewing other women being objectified, social media influencer content can be one of the important triggers of self-objectification. The users who follow these types of social media influencers are mostly young women. They are more vulnerable to unattainable body ideals on social media and often comply with narrowly defined social beauty standards. In summary, when women are exposed to body ideals through their media consumption, their attentions may be led to monitor and assess observable body characteristics, triggering negative perceptions or behavioral intentions toward their body. Therefore, this study proposes that women’s social media influencer viewing frequency should be associated with their degree of self-objectification and self-objectification should mediate the relation between influencer viewing and intentions to change appearance.

The extent to which social media influencer viewing can affect women’s intentions to change appearance may also depend on the degree to which they make social comparisons between themselves and others. Social comparison can be defined as a process through which individuals seek information and evaluate themselves in comparison with others (Festinger, 1954). Previous studies examining the traditional media effects of exposure to thin-ideals on TVs and magazines on body dissatisfaction, as well as several recent studies examining the association between social media use and body image concerns adopt social comparison as an explanatory mechanism (Want, 2009; Fardouly et al., 2018; Saiphoo and Vahedi, 2019).

Social comparison can both refer to a contextual process that describes the extent and the direction to which a person makes comparisons between themselves and others (*state social comparison*), or a stable motivation for one to position him or herself in social circles (*trait social comparison*) (Want, 2009). State social comparison can be activated when exposed to idealized images and has been identified as a mediator of exposure effects of idealized images on body dissatisfaction (Betz et al., 2019). However, individual differences may exist in terms of the extent to which one makes a comparison between the self and others. Trait social comparison describes the general tendency to compare oneself to others and has been linked with body dissatisfaction (Myers and Crowther, 2009; Betz et al., 2019). For example, an experimental study finds that state social comparison mediated the body ideal exposure effects on body surveillance and body appreciation, and trait social comparison moderated the association between viewing curvy ideal body and state social comparison (Betz et al., 2019). Similarly, we also conceptualize social comparison as a stable trait, or *social comparison tendencies* and investigate how this trait moderates the proposed relationship between social media influencer viewing and intentions to change appearance. We conceptualize and operationalize self-objectification on state-level and social comparison on trait level. Therefore, self-objectification is treated as the mediator while social comparison tendencies as the moderator.

Considering that the focus of this study is on social media and influencer viewing, we specifically contextualize social comparison tendencies in terms of short video watching. Therefore, when viewing content shared by social media influencers, women who have a higher level of social comparison tendencies (i.e., trait-level social comparison) while watching short videos would pay more attention to the appearance of the influencers and themselves. For women who have a lower level of social comparison tendencies, they may be less aware of the appearance of the influencers and themselves. For female TikTok users, their social comparison tendencies may intensify the association between social media influencer viewing and intentions to change appearance.

### The Current Study

In summary, prior studies mainly focused on the general social media use frequency or specific use of Facebook and Instagram and their associations with body image concerns or body dissatisfaction. Less is known about the impact of exposure to emerging platforms popular among young women and other targets such as social media influencers acting as body ideals. Furthermore, this study also seeks to extend previous research by adopting behavioral intentions as the outcome variable. Previous studies mainly adopt psychological states such as body image concerns and body dissatisfaction as outcome variables due to their impacts on individuals’ psychological and physical well-being. However, intentions to change appearance should also be considered to extend research on body image as they are moderately associated with individuals’ actual behaviors. For example, one meta-analysis on 22 published studies reports that behavioral intention, in general, was moderately associated with actual dieting behavior, *r* = 0.47 (McDermott et al., 2015).

The current study aimed to extend previous studies examining the association between social media use and body dissatisfaction by focusing on one specific type of social media use, social media influencer viewing, and its association with intentions to change appearance **(H1)**. Specifically, we tested whether this proposed association was mediated by individuals’ self-objectification **(H2)**. Furthermore, we examined whether participants’ social comparison tendencies would moderate the association between social media influencer viewing and intentions to change appearance **(H3)**. For women who have a higher level of social comparison tendencies, the association between social media influencer viewing and intentions to change appearance would be stronger, compared to women who have a lower level of social comparison tendencies.

## Methods

### Participants and Procedure

A cross-sectional survey was conducted in collaboration with TikTok, China. The survey received ethical approval from the Internal Review Board from a large central university in Beijing, China. The online survey questionnaire was designed by the researchers of the current study and sent to TikTok, China. TikTok, China then “pushed” (sent a notification within the TikTok APP to users’ mobile phone) the online questionnaire to target users (registered at least 14 days and self-reported female users with the age ranging from 18 to 60, received a push notification on TikTok APP and at least clicked the push notification one time within 30 days, and recent 90-days active time larger than 60 days) of the TikTok mobile application in China. The invitation of survey participation was sent to 60,000 users who fit the inclusion criteria and a total number of 7,015 users finished and submitted their responses.

Participants were first asked about their social media influencer viewing frequency. Then they were asked about their social comparison tendencies while watching short videos on social media, self-objectification, and intentions to change appearance. Lastly, they were surveyed about their demographic information including their age, height, weight, marital status, education level and employment status. Although participants were recruited on TikTok mobile application, the survey questions did not only pertain to TikTok-related usage. A sample of 7,015 adult female TikTok users with a mean age of 30.42 (*SD* = 7.73) and a mean body mass index (BMI) of 21.23 (*SD* = 3.21) participated in this online survey. About 48.60% of the participants reported to be single (*n* = 3409), 39.86% were married (*n* = 2796), followed by 8.34% devoiced (*n* = 585), 2.89% separated (*n* = 203), and 0.31% widowed (*n* = 22). In terms of education level, 53% received less than high school level education (*n* = 3718), 44.99% received the associate or bachelor’s degree (*n* = 3156), and 2.01% received graduate-level education (*n* = 141). In terms of the employment status, 41.54% of the participants reported to be fully employed (*n* = 2914), 34.71% self-employed (*n* = 2435), followed by 19.19% unemployed or unable to work (*n* = 1346), 4.01% working part-time (*n* = 281), and 0.56% retired (*n* = 39).

Considering that the survey was conducted in China and on Chinese participants, the original scales composed in the English language were translated to Chinese by a research assistant who is proficient in both English and Chinese. To ensure the accuracy and appropriateness of the translation, another research assistant who is also proficient in both languages independently back-translated the Chinese questionnaire into English. The back-translated version was then compared with the original English version, and any deviation in meanings was addressed by revising and re-translating the Chinese questionnaire.

### Measures

#### Social Media Influencer Viewing

Participants’ social media influencer viewing frequency was measured using three items on a five-point Likert-type scale ranging from “never = 1” to “always = 5.” Sample items included “how frequently do you watch selfies posted by influencers on social media” and “how frequently do you watch live streaming of influencers on social media.” The three items showed good reliability (α = 0.84).

#### Social Comparison Tendencies While Watching Short Videos

Participants’ frequency to engage in social comparisons while watching short videos were measured by three items on a five-point Likert-type scale ranging from “never = 1” to “always = 5.” These items were adapted from the Physical Appearance Comparison Scale-Revised (PACS-R) and showed good reliability (Schaefer and Thompson, 2014), α = 0.92. Sample items included “while watching short videos, I would compare my appearance with others” and “while watching short videos, I would compare my body with others.”

#### Self-Objectification

Participants’ self-objectification was measured by six items on a five-point Likert scale ranging from “strongly disagree = 1” to “strongly agree = 5.” These items were adapted from the Self-objectification Beliefs and Behaviors Scale (SOBBS) and showed acceptable reliability (Lindner and Tantleff-Dunn, 2017), α = 0.79. Sample items included “looking attractive to others is more important to me than being happy with who I am on the inside” and “how slim my body looks says something about who I am as a person.”

#### Intentions to Change Appearance

Participants’ intentions to change their appearance were measured by four items on a five-point Likert scale ranging from “strongly disagree = 1” to “strongly agree = 5.” These items were adapted from the Acceptance of Cosmetic Surgery Scale (ACSS) and showed good reliability (Henderson-King and Henderson-King, 2005), α = 0.88. Sample items included “I had the idea that I needed to get in shape because of something I saw on social media,” and “I thought I needed to improve my physical appearance because of something I read on social media.” All survey items can be found in the Supplementary Material.

### Data Analysis

In order to test the association between social media influencer viewing and intentions to change appearance, hierarchical linear regression analysis was performed controlling for participants’ age and BMI. Previous studies on body image often found positive associations between individuals’ BMI and body dissatisfaction (e.g., Tiggemann and McGill, 2004). BMI has been measured as a covariate in body dissatisfaction studies to show the discrepancy between participants’ actual and perceived bodies (Grabe and Hyde, 2006) and was also found to be positively associated with individuals’ planned weight-loss behaviors (Pan and Peña, 2021). Therefore, all hypotheses testing controlled for participants’ BMI and age. In the first regression model, participants’ age and BMI were entered as predicting variables and intentions to change appearance as the outcome variable. In the second regression model, participants’ social media influencer viewing was entered as the predicting variable and intentions to change appearance as the outcome variable. Model 4 of PROCESS macro in SPSS was used to test the mediation of self-objectification and Model 1 of PROCESS macro was used to test the moderation of social comparison tendencies (Hayes, 2017).

## Results

Descriptive statistics for TikTok use and influencer viewing were displayed in Table 1. Pearson correlations on all main variables were included in Table 2.

**TABLE 1:** Means and standard deviations of key variables.

	*M*	SD	Minimum	Maximum	Range
Age	30.36	7.69	19	58	39
BMI	21.22	3.20	15.52	38.22	22.70
Social media influencer viewing	2.99	0.95	1	5	4
Social comparison tendencies	2.54	1.07	1	5	4
Self-objectification	3.28	0.81	1	5	4
Intentions to change appearance	3.06	1.17	1	5	4

*Social media influencer viewing range = 1–5; Social comparison tendencies range = 1–5; Self-objectification range = 1–5; Intentions to change appearance range = 1–5.*

**TABLE 2:** Zero-order correlations among key variables.

Variables	1	2	3	4
1. Social media influencer viewing	1			
2. Social comparison tendencies	0.307**	1		
3. Self-objectification	0.152**	0.424**	1	
4. Intentions to change appearance	0.202**	0.444**	0.434**	1

**p < 0.01.

### Relation Between Influencer Viewing and Intentions to Change Appearance

In order to perform linear regression analysis, the data were first analyzed to meet the assumptions. The Durbin-Watson statistic was 1.96 which is between 1.5 and 2.5 and therefore, the data was not autocorrelated. For the collinearity statistics, in the first regression model, the tolerance is 0.997 and VIF = 1.002. In the second regression model, the tolerance is 0.997 and VIF = 1.003. These collinearity statistics indicated that the variables in the regression did not have multicollinearity.

Hierarchical regression analysis showed that participants’ age (*M* = 30.42, *SD* = 7.73) negatively predicted their intentions to change appearance (*M* = 3.05, *SD* = 1.16), β = −0.12, *t*(7012) = −10.21, *p* < 0.001, and their BMI (*M* = 21.23, *SD* = 3.21) positively predicted their intentions to change appearance (*M* = 3.05, *SD* = 1.16), β = 0.20, *t*(7012) = 17.1, *p* < 0.001. Controlling for participants’ age and BMI, social media influencer viewing positively (*M* = 2.99, *SD* = 0.95) predicted participants’ intentions to change appearance (*M* = 3.05, *SD* = 1.16), β = 0.20, *t*(7011) = 17.36, *p* < 0.001. Social media influencer viewing explained a significant proportion of the variance in their intentions to change appearance, Δ*R*^2^ = 0.04, *F*(1,7011) = 301.32, *p* < 0.001. Although a coefficient of determination (*R*^2^) of 0.04 is considered as small, the results can still have implications to study the association between social media influencer viewing and intentions to change appearance.

### Mediation of Self-Objectification

**FIGURE 1:**
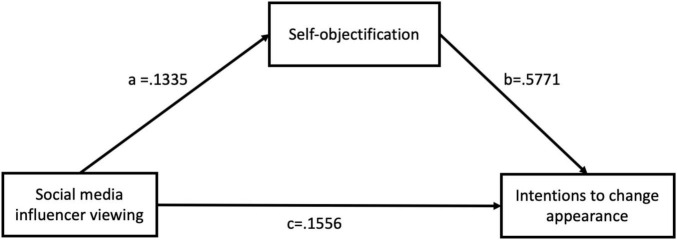
Social media influencer viewing predicting intentions to change appearance through self-objectification as the mediator.

In order to test for the role of self-objectification in mediating the association between social media influencer viewing and intentions to change appearance, PROCESS macro in SPSS was used (model 4) (Hayes, 2017). Participants’ social media influencer viewing frequency was entered as the predictor, self-objectification as the mediator, intentions to change appearance as the outcome variable, and participants’ age and BMI were entered as covariates. The direct effect of social media influencer viewing on intentions to change appearance was significant, *b* = 0.1556, *SE* = 0.0132, *p* < 0.001, CI = [0.1297, 0.1815]. The indirect effect of social media influencer viewing on intentions to change appearance mediated through self-objectification was significant (*b* = 0.0770, *SE* = 0.0066, 95% CI = [0.0467, 0.0666]) and confirmed by bootstrapping test based on 5,000 resamples. The mediation model can be found in Figure 1.

### Moderation of Social Comparison Tendencies

**FIGURE 2:**
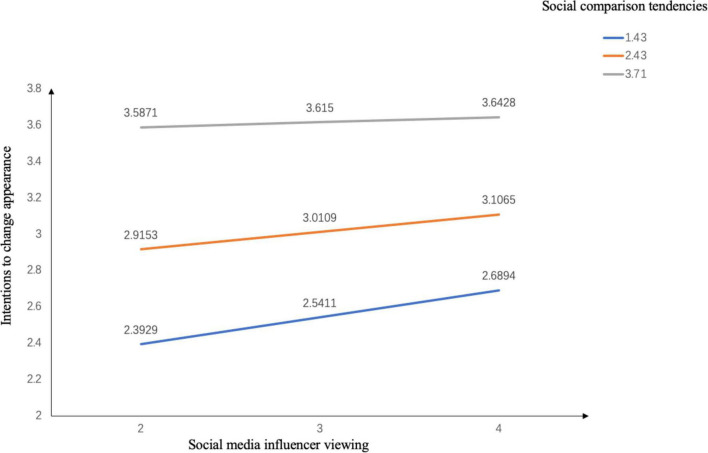
Social comparison tendencies moderating the association between social media influencer viewing and intentions to change appearance.

In order to test for the role of social comparison tendencies in moderating the association between social media influencer viewing and intentions to change appearance, model 1 of PROCESS macro in SPSS was used (Hayes, 2017). Participants’ social media influencer viewing frequency was entered as the predictor, social comparison tendencies as the moderator, intentions to change appearance as the outcome variable, and participants’ age and BMI were entered as covariates. The interaction between social media influencer viewing and social comparison tendencies was significant, *b* = −0.0527, *t* = −4.78, *p* < 0.001. The conditional effect of social media influencer viewing on intentions to change appearance when social comparison tendencies are at 1 SD lower than the mean value was significant, *b* = 0.1483, *SE* = 0.0184, *p* < 0.001. The proposed conditional effect when social comparison tendencies are at the mean value was significant, *b* = 0.0956, *SE* = 0.0135, *p* < 0.001. The proposed conditional effect when social comparison tendencies are at 1 SD higher than the mean value was not significant, *b* = 0.0279, *SE* = 0.0184, *p* = 0.13. The conditional effects are illustrated in Figure 2. Although the interaction between social media influencer viewing and social comparison tendencies was significant, the observed interaction effect was the opposite of H3.

## Discussion

Though previous studies have examined the association between social media use and body image concerns, the present study focused on social media influencer viewing as one unique type of social media use due to its growing popularity among young people. Through a large-scale survey conducted on current adult female users of TikTok in China, the current study found a positive association between users’ influencer viewing frequency and their intentions to change appearance. This result was congruent with previous studies examining the exposure effects of body ideal portrayals on body image concerns both on traditional and social media (Grabe et al., 2008; Baker et al., 2019; Saiphoo and Vahedi, 2019). Short video APPs such as TikTok were featured with celebrities, Internet celebrities, and social media influencers and they provided abundant technological affordances such as filters, special effects, and other types of photo and video editing functions (e.g., acne dermabrasion, face-lift, airbrushing, whitening, eye zoom, etc.) to create an idealized self-presentation. This type of content created on short video APPs may promote an unattainable standard of beauty.

Furthermore, the results showed that the association between influencer viewing and intentions to change appearance was further mediated by female TikTok users’ self-objectification. This pattern has also been observed by previous literature focusing on the association between sexually objectifying TV shows, music videos, and magazines and women’s self-perceptions (Aubrey, 2006). One study also reported a positive association between Facebook photo-related activity and body image disturbance (Meier and Gray, 2014). Considering the nature of the content created by social media influencers on TikTok and their marketing value and profit model, the results of the current study indicated that influencer viewing may promote women to self-objectify their own bodies and appearances. Seeing their own body appearance as “objects,” female TikTok users may resort to weight-loss or plastic surgeries to change their own appearance.

However, contrary to what we have predicted, the results showed that the proposed moderating role of social comparison tendencies was in the opposite direction. The association between influencer viewing and intentions to change appearance was stronger for users who have lower social comparison tendencies compared to the ones who have higher social comparison tendencies. A similar trend has also been observed in several experimental studies. When participants were not told to engage in social comparisons, they reported higher body dissatisfaction compared to participants who were told to engage in social comparisons (Want, 2009). One possible explanation of this opposite association would be that individuals who have higher social comparison tendencies may be more aware of the detrimental effects of body ideals and social comparison. They can “undo” the unfavorable social comparison by consciously processing and adjusting their mental status (Gilbert et al., 1995; Pan and Peña, 2021).

In terms of demographic variables, age was negatively associated with female TikTok users’ intentions to change their appearance. One possible explanation of this finding is that although body dissatisfaction among women seems to remain constant across the lifespan, the importance of body shape, weight and appearance may decline over time (Grogan, 2016). Meanwhile, cognitive control developed along age could help to protect women against the influence of body dissatisfaction (Webster and Tiggemann, 2003). The results also showed that among female TikTok users, higher BMI was linked to a higher intention to change appearance. This could be explained by empirical evidence suggesting that BMI was positively associated with body dissatisfaction among women (Ålgars et al., 2009; Heider et al., 2015).

### Practical Implications

Although TikTok aims to “encourage users to share their passion and creative expression through their videos,” negative outcomes toward individuals’ body image triggered by idealized influencer content should not be overlooked. Considering that an important motivation for content creation on TikTok is for profit, influencers on social media tend to cater to existing appearance norms rather than promoting acceptance and respect for realistic body image. Social media influencers’ self-objectified propensity is also irrelevant to foster body appreciation among their followers. Given the growing TikTok usage among the young generation especially young women, they may encounter a wide range of unattainable body ideals not only as short video audiences but also as potential consumers of influencers. Exposure to idealized body image portrayed by influencers may relate to a variety of body image concerns or risky behaviors to change their appearance. Media literacy programs should be designed to warn teenagers and young adults about the potential negative outcomes of short video APP use on body image. The observed opposite moderation effect should also inform the design of public health campaigns that aim to cultivate body appreciation.

One caveat regarding the association between social media influencer viewing and intentions to change appearance lies in the trivial effect size. The observed small but statistically significant effect size could be attributed to the relatively large sample size. In study designs, a larger sample size was usually preferred compared to smaller ones in order to extend the generalizability of the findings. However, this may introduce higher variability and may be difficult to control other relevant factors contributing to the outcome variables (Ferguson, 2016). The results of the current study should be interpreted carefully since the observed results could be due to the unique characteristics of the TikTok user samples or the specific questions we have adopted in the survey. Caution should be given when evaluating the practical implications of the current study.

### Limitations

This study has several limitations. The first limitation pertains to the cross-sectional survey design of the current study. Although a relatively large sample size was achieved, the correlational nature cannot valid any causal inferences. Future studies should adopt experiments or ecological momentary assessment to explore causal relations. Second, all survey responses collected from female TikTok users are self-report, therefore, the data may suffer from inaccurate recall or potential social desirability bias. The third limitation pertains to the measurement. Considering that the focus of the current study is a unique type of social media use, the survey items used to measure intentions to change appearance only pertain to the influence of social media. However, participants could form intentions to change appearance based on other factors such as their pre-existing body satisfaction and body esteem instead of social media. This could generate noise and interfere with the interpretation of the results. Future studies could design more detailed survey items to measure intentions to change appearance and control for other factors contributing to behavioral intentions. Lastly, social media influencer viewing only accounted for a small portion of the variance in intentions to change appearance. Although we have observed a statistically significant association between social media influencer viewing and intentions to change appearance, caution should be given when considering the practical implications and the interpretation of the results. Future studies should continue to identify other cultural, societal, and psychological factors contributing to intentions to change appearance.

## Data Availability Statement

The raw data supporting the conclusions of this article will be made available by the authors, without undue reservation.

## Ethics Statement

The studies involving human participants were reviewed and approved by the Institutional Review Board, School of Journalism and Communication, Renmin University of China. The patients/participants provided their written informed consent to participate in this study.

## Author Contributions

WP and ZT contributed to the conception and design of the study. ZT organized the survey. WP and ZM performed the statistical analysis and wrote the first draft of the manuscript. All authors worked on revisions of the manuscript and performed the additional statistical analysis, wrote the sections of the manuscript, contributed to manuscript revision, read, and approved the submitted version.

## Conflict of Interest

The authors declare that the research was conducted in the absence of any commercial or financial relationships that could be construed as a potential conflict of interest.

## Publisher’s Note

All claims expressed in this article are solely those of the authors and do not necessarily represent those of their affiliated organizations, or those of the publisher, the editors and the reviewers. Any product that may be evaluated in this article, or claim that may be made by its manufacturer, is not guaranteed or endorsed by the publisher.
