# Supplementary material for: Social Media Influencer Viewing and Intentions to Change Appearance: A Large Scale Cross-Sectional Survey on Female Social Media Users in China
Source: Front Psychol. 2022 Apr 8;13:846390. doi: 10.3389/fpsyg.2022.846390 (PMC9024235; doi:10.3389/fpsyg.2022.846390)
Supplement: Supplementary file 1 [file Table_1.DOCX]

Survey items

| Variables | Items |
| --- | --- |
| Social media influencer viewing | 1. How frequently do you watch content generated by influencers on social media? |
|  | 2. How frequently do you watch selfies posted by influencers on social media? |
|  | 3. How frequently do you watch live streaming of influencers on social media? |
|  |  |
| Social comparison tendencies while watching short videos | 1. While watching short videos, I would compare my appearances with others. |
|  | 2. While watching short videos, I would compare my clothes with others. |
|  | 3. While watching short videos, I would compare my body with others. |
| Self-objectification | 1. Looking attractive to others is more important to me than being happy with who I am on the inside. |
|  | 2. How slim my body looks says something about who I am as a person. |
|  | 3. I try to imagine what my body looks like to other people. |
|  | 4. I choose specific clothing or accessories based on whether they make my appearance attractive to others. |
|  | 5. When I look in the mirror, I notice areas of my appearance that I think others will view critically. |
|  | 6. I consider how my body will look to others in the clothing I am wearing. |
| Intentions to change appearance | 1. I had the idea that I needed to get in shape because of something I saw on social media. |
|  | 2. I thought about getting thinner because of something I saw on social media. |
|  | 3. I thought I needed to improve my physical appearance because of something I read on social media. |
|  | 4. I considered that I needed to lose weight because of something I saw on social media. |
